# Report of an Extremely Rare Case of BRCA-Positive Head and Neck Cancer: A New Disease on the Horizon?

**DOI:** 10.7759/cureus.78175

**Published:** 2025-01-29

**Authors:** Sadanand M Karandikar, Himanshi Joon, Shailesh Puntambekar

**Affiliations:** 1 Medical Oncology, Ruby Hall Clinic, Pune, IND; 2 Medical Oncology, Galaxy Care Hospital, Pune, IND; 3 Obstetrics and Gynecology, Galaxy Care Hospital, Pune, IND

**Keywords:** brca mutation, cup with metastatic scc neck nodes, hnscc, liquid biopsy, parp inhibitors, platinum-based chemotherapy

## Abstract

Cancer of unknown primary (CUP) with metastatic squamous cell carcinoma (SCC) to the neck nodes is rare and represents <5% of all head and neck malignancies. The prognosis is poor; hence, there is a need for early comprehensive diagnostic evaluation and management. A BRCA mutation is rarely seen in patients with head and neck squamous cell carcinoma (HNSCC) with an incidence of only 5-10%. We hereby present a rare case report of CUP with metastatic neck nodes (SCC), unveiling a BRCA mutation on liquid biopsy. The patient responded very well with platinum-based cytotoxic chemotherapy followed by maintenance poly adenosine diphosphate ribose polymerase inhibitors (PARPi). This is a first-of-its-kind case in the head and neck region, especially in this part of the world, where tobacco-related head and neck cancer catches all the attention over human papillomavirus (HPV) positivity and other risk factors.

## Introduction

Squamous cell carcinoma (SCC) metastatic to cervical lymph nodes from an unknown primary site represents <5% of all head and neck malignancies [1]. These patients pose a unique challenge to oncologists, as careful assessment and evaluation are the key to identifying the underlying primary site and subsequently directing us to appropriate treatment strategies [2]. In recent years, the importance and recognition of germline oncogenic mutations have grown, contributing to the pathogenesis of various cancers. Among these, Breast Cancer Susceptibility Genes 1 & 2 (BRCA 1 & 2) have emerged and gained utmost importance in the expanding spectrum of malignancies including breast, ovarian, pancreatic, and prostate cancer and those of the head and neck as well [3-5]. We hereby present an extremely rare case of CUP with metastatic SCC to the neck nodes with the BRCA 1 mutation, treated with first-line chemotherapy and now on maintenance with poly adenosine diphosphate ribose polymerase (PARP) inhibitors.

## Case presentation

**Figure 1:**
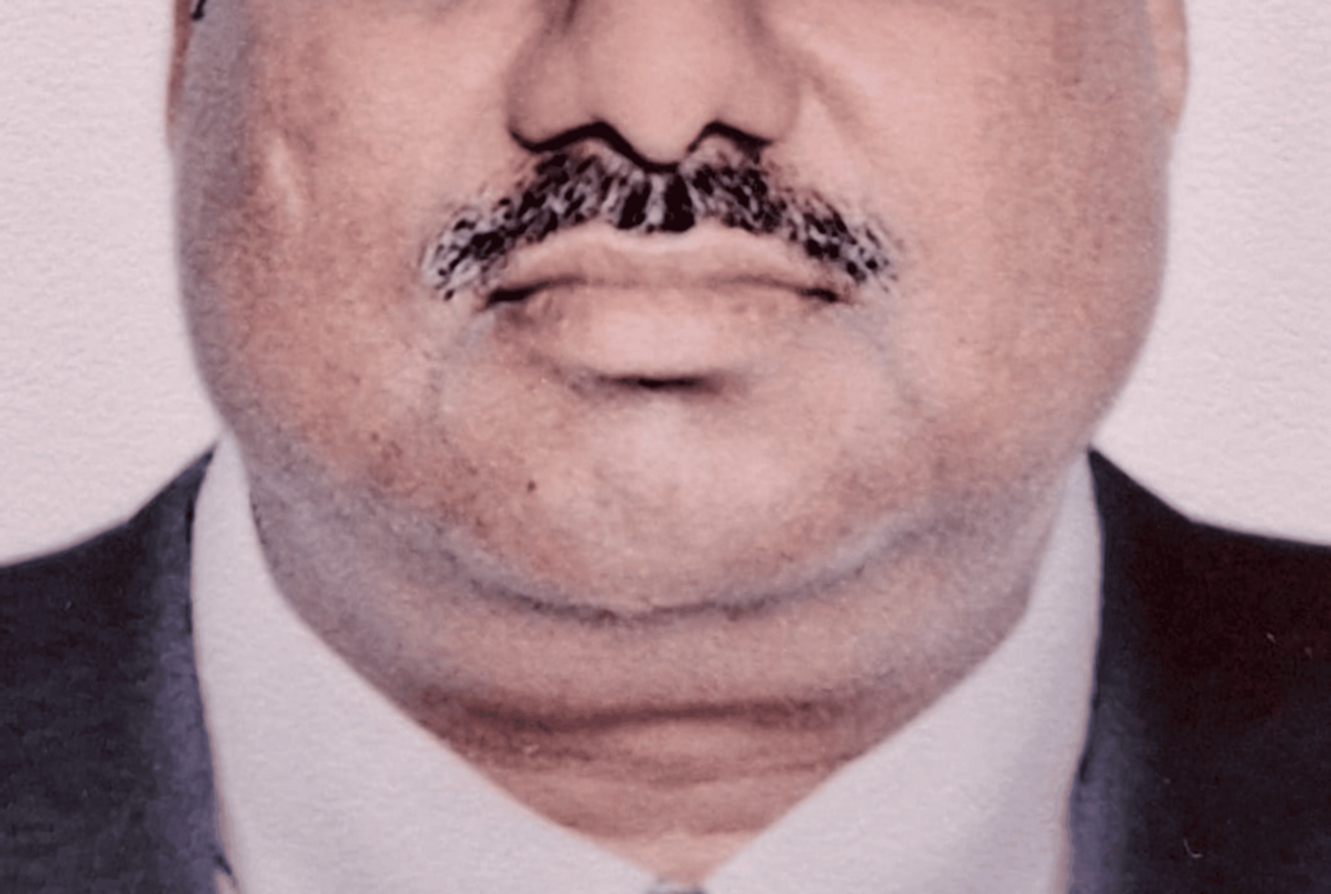
Patient presenting with noticeable bilateral neck swelling in 2023

**Figure 2:**
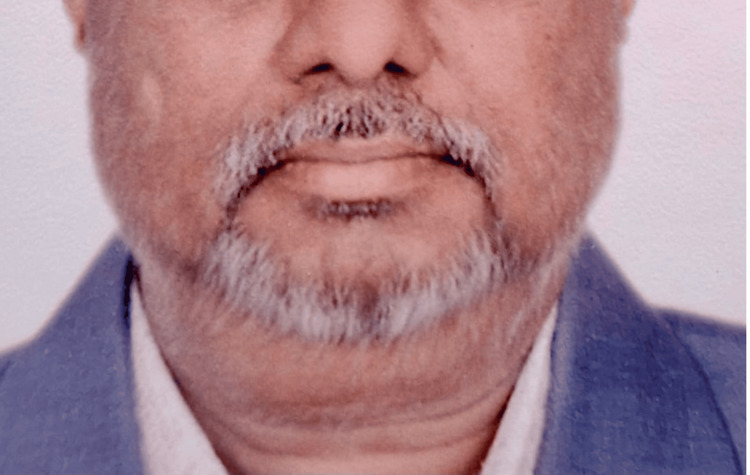
Partial response after six cycles of platinum chemotherapy with substantially decreased neck swelling is seen.

A 60-year-old, male, Asian patient presented in September 2023 with gradual and painless bilateral neck swelling for two months (Figure 1). No “B” symptoms or complaints related to the upper aerodigestive tract were associated. A careful physical examination revealed matted and enlarged bilateral neck nodes, but no lesion was seen on the naked eye in the oral cavity/oropharynx/nasopharynx and larynx on indirect laryngoscopy (IDL). A complete diagnostic workup was done, and triple endoscopy of the upper aerodigestive tract and whole body fludeoxyglucose-18 (FDG) positron emission tomography (PET)-computed tomography (CT) failed to reveal any primary site lesion. CBC, liver function test (LFT), renal function test (RFT), electrolytes, and tumor markers like carcinoembryonic antigen (CEA), prostate-specific antigen (PSA), alpha-fetoprotein (AFP), human chorionic gonadotropin (HCG), and lactate dehydrogenase (LDH) were all within normal biological values. Hence, the diagnosis of cancer of unknown primary (CUP) was made and an excision biopsy of a cervical lymph node was planned and performed, which came out as metastatic squamous cell carcinoma negative for p16, ruling out lymphoproliferative disorders as well. The immunohistochemistry (IHC) report was as follows: CK5/6 positive, p63 and p40 positive, negative for CK7, CK 20, TTF 1, NSE, S100, and Vimentin. Based on this, the patient was started on intravenous chemotherapy with modified docetaxel, cisplatin, and 5-FU (5-fluorouracil) (DCF) protocol in standard doses consisting of nab-paclitaxel, cisplatin, and 5FU to lower disease burden; meanwhile, a 523 gene panel liquid biopsy test (Tempus, Chicago, IL, US) was sent for further clarification on the disease biology (Table 1). A drastic clinical response was achieved and with tolerable toxicity, six cycles were given (Figure 2).

**Table 1:** Liquid biopsy showing genomic variants and immunotherapy markers

GENOMIC VARIANTS	
Potentially Actionable	Variant Allele Fraction
BRCA 1, p.E23fs frameshift - LOF	45.1%
PIK3CA p.R93W missense variant (exon 1)	0.2%
Biologically Relevant	
TP53 p.R283P Missense variant - LOF	0.5%
MYC Copy number gain	-
ctDNA tumor fraction	0.4%
IMMUNOTHERAPY MARKERS	
Blood tumor mutational burden (bTMB)	5.0 m/MB

Programmed death ligand (PD-L1) on tissue blocks was strongly positive with CPS 50%.

**Figure 3:**
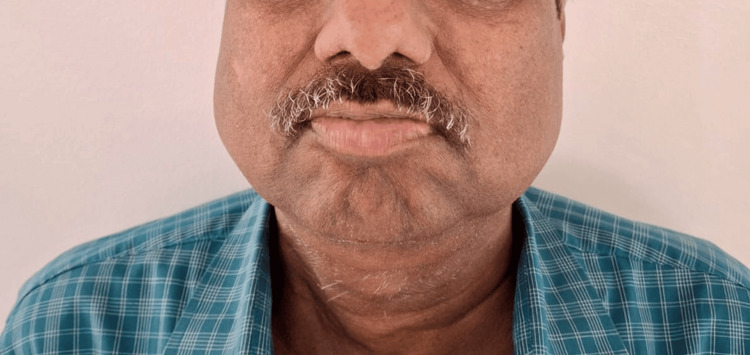
Further response and reversal of chemotherapy toxicity seen after six months of treatment with rucaparib

On follow-up imaging for response assessment after six cycles of chemotherapy, partial response with >50% disease control on FDG PET-CT was achieved; hence, he was started on PARP inhibitors with Tab. Olaparib 300 mg BID in view of his BRCA 1 positivity. This was poorly tolerated, and the patient developed severe cytopenia, which led to drug discontinuation and hospitalization for supportive management. On recovery, rucaparib was substituted with a 50% dose reduction at 300 mg BID 12 hours apart. This dose was well-tolerated, and the patient remains in disease control to date. Dose escalation was carefully done, and he is now well-controlled on Tab. Rucaparib 600 mg in the morning and 300 mg in the evening for the past six months (Figure 3).

We have reserved the options of radiotherapy, immunotherapy, and other targeted therapies for the future in eventual relapse/disease progression, as we are evaluating the response of PARP inhibitors in such rare cases.

## Discussion

Many head and neck cancer patients present clinically with cervical lymphatic metastasis as the only complaint; hence, they are labeled as “unknown primary”, but the majority have a primary established on careful physical examination, endoscopic examination, and imaging [2]. Only after extensive evaluation, patients like ours can be categorized as cancer of unknown primary (CUP). Metastatic squamous cell carcinoma of cervical lymph nodes occurs in only 1-7% of new head and neck cancer cases, which decreases to less than 3% after extensive workup [5-8]. The prognosis of metastatic patients remains poor with a 5-year OS rate of 10-15%. The standard treatment of CUP with metastatic SCC neck nodes included chemotherapy, sequential chemo-radiotherapy/ concurrent chemo-radiotherapy, immunotherapy, and salvage neck dissection in isolated lymph nodal group relapse cases [9]. BRCA mutation in males is known to confer a higher risk for the development of various tumors; however, its incidence in head and neck cancers is low and no literature is available, mentioning its burden in CUP with metastatic cervical lymph node (LN) SCC like our case. This is a unique and rare presentation and is “one of its kind,” to the best of our knowledge.

The possible association between BRCA gene mutations and head and neck cancers is not well-established. Shen TK et al. observed that the rate of 3 of 5754 cases (0.052%) of head and neck cancers in BRCA mutation-positive probands and likely carriers is significantly higher than the background incidence rate of 3 per 100 000 (0.003%) per year (P < .001) [10]. Somatic mutations in BRCA1 are seen in only 6% and BRCA 2 in 7% of head and neck squamous cell carcinoma (HNSCC) patients, indicating the multistep process involved in tumorigenesis. Genetic analysis suggests that homologous recombination deficiency (HRD) and polymorphisms in the single strand breaks (SSB) repair system are common in HNSCC, which is also thought to lead to the upregulation of PD-L1 as seen in our case [11].

The molecular profiling of HNSCC patient samples has identified BRCA1 and BRCA2 mutations with a frequency of 5.75% and 9.2%, respectively, which seem to co-exist with TP53 and PI3KCA pathway mutations, as seen in our patient [12]. However, the clinical significance of most detected variants remains to be determined. Mutations in BRCA1 and BRAC2 genes create a defect in HRD, making cells harboring these mutations susceptible to DNA-damaging agents, such as cisplatin, which leads to permanent cell damage and death, thereby making us understand why our patient responded so well to systemic chemotherapy. Polyadenosine diphosphate ribose polymerase inhibitors (PARPi) lead to increased DNA damage and increased inflammation, leading to infiltration by immune cells, causing an effective immune reaction clearing the remaining tumor cells [11]. Hence, our decision to optimize our patient’s treatment with olaparib/rucaparib with an attempt to reduce the relapse rate risk by blocking the DNA repair mechanism after treatment with systemic chemotherapy. It is still too early to know the response rate (RR)/progression-free survival (PFS) and impact on overall survival (OS); however, the authors hope that our rare case report will lead to more individualized trials and ultimately culminate in a better understanding of these BRCA genes involved in other solid organ malignancies than carcinoma breast, ovary, prostate and pancreas, leading to revolutionized treatment modalities for BRCA-positive patients in HNSCC/CUP with neck nodes SCC.

## Conclusions

A BRCA mutation is seen in only 5-10% of HNSCC but no case has been reported with the mutation seen in CUP with SCC metastatic to the neck nodes yet; hence, our attempt to bring notice to the vast tumorigenic potential of this gene. This case report also underlines the criticality and importance of molecular genetic testing of such patients at disease presentation for a better understanding of disease biology leading to more accurate lines of treatment. Fixed guidelines on the management of such cases are not yet available. The authors propose that platinum-based systemic chemotherapy with a standard dose of paclitaxel, cisplatin, and 5FU to downstage the disease burden, followed by maintenance PARPi, is an effective way to treat these malignancies. This case might be the first of its kind in the head and neck region, especially in this part of the world where tobacco-related head and neck cancer catches all the attention over HPV positivity and other risk factors. Although it’s difficult to postulate this based on a single case report, the authors wish to coin a separate entity as “BRCA-positive head and neck disease” that needs specific categorization and annotation for the further development of management guidelines along with an understanding of the role of PARPi. The authors also hope that this report of a rare case report will lead to more individualized studies and a better understanding of the BRCA genes involved in other solid organ malignancies with treatment guidelines for BRCA-positive patients in HNSCC/CUP with SCC of the neck nodes.
